# A controlled clinical study on efficacy and safety of periocular triamcinolone acetonide injection for treating ocular myasthenia gravis

**DOI:** 10.1186/s12886-024-03313-z

**Published:** 2024-01-22

**Authors:** Minghua Shi, Zhuneng Lu, Aijiao Qin, Jing Cheng, Simin Chen, Yiqiao Xing

**Affiliations:** 1 Department of Opthalmology, Aier Eye Hospital of Wuhan University, No. 481, Zhongshan Road, Wuchang District, Wuhan, 430060 China; 2 Department of Neurology, RenMin Hospital of Wuhan University, Wuhan, China

**Keywords:** Ocular myasthenia gravis, Ptosis, Ophthalmoplegia, Peribulbar injection, Triamcinolone acetonide

## Abstract

**Objective:**

To evaluate the efficacy and safety of peribulbar triamcinolone acetonide injection for treating ocular myasthenia gravis (OMG), with a comparison of traditional oral drug therapy.

**Methods:**

A total of 22 patients with OMG who received periocular triamcinolone acetonide injection (initially 20 mg weekly, then once per month later if symptoms were improved) from July 2019 to July 2022 were evaluated by a comparison of symptom degree before and after treatment. Adverse reactions were also monitored during the period of treatment. The period of follow-up was more than 6 months. Additionally, a comparison of the treatment efficacy between this periocular injection and traditional oral administration was performed in OMG patients.

**Results:**

After 4 weeks of treatment, the degree of ptosis in OMG patients decreased to -3.00 ± 0.69, compared to the value (-0.86 ± 1.32) before treatment. The degree of ophthalmoplegia also decreased from 3.12 ± 0.72 to 0.86 ± 0.88 (*P* < 0.001) after treatment. The achievement rates of minimal manifestations status (MMS)for ptosis and ophthalmoplegia after 4 week-treatment were 86.3% and 75%, respectively, while they were 50% and 30% in patients with traditional oral administration. There was statistically significant difference only in MMS (rather than symptom relief rate and generalization conversion rate) between two groups. No serious complications (except for intraorbital hematoma) were found in OMG patients during the treatment period.

**Conclusion:**

Repeated peribulbar injection of triamcinolone acetonide can effectively alleviate the initial symptoms of OMG patients. However, the evaluation of its long-term efficacy is still needed.

**Clinical Trial Registry:**

This study has been clinically registered by Chinese Clinical Trial Registry (ChiCTR), first trial registration date:05/07/2019, registration number: ChiCTR1900024285.

## Background

Myasthenia Gravis (MG) is a neuromuscular disorder that occurs when the body’s immune system attacks the postsynaptic region of the neuromuscular junction. The incidence rate of MG is low, with only 10 to 30 cases per million person-years [1–3]. The standard treatment for MG is oral cholinesterase inhibitors and/or corticosteroids, while immunosuppressive therapies are recommended for MG patients with poor response to the former [4, 5]. However, the treatment outcomes of systemic medication for MG have been poor, with less than 30% complete remission rate [6, 7] and 10–40% of patients developing generalized myasthenia gravis (GMG) [6–13]. Additionally, the side effects of systemic medication may cause difficulty in treatment.

The extraocular muscle (EOM) differs from other skeletal muscles in anatomy, antigen structure, and complement activation mechanism, making it more prone to autoimmune diseases such as Thyroid Associated Ophthalmopathy (TAO) and MG [14–17]. About 85% of MG patients present with extraocular symptoms such as ptosis and/or diplopia, and half of all cases are only limited to the ocular area [7]. Previous studies have shown that periocular injection of triamcinolone acetonide is more effective for TAO than systemic oral corticosteroids, especially for patients in the acute phase, because TAO is mainly caused by inflammation of the EOM [18–20]. Moreover, small-scale case studies have demonstrated that local use of corticosteroids can rapidly relieve ocular symptoms in OMG patients [21]. This study aims to evaluate the efficacy and side effects of peribulbar triamcinolone acetonide treatment for OMG and compare it with conventional oral pharmacotherapy.

## Patients and methods

### Patients enrolled in the study

This study enrolled all patients with ocular myasthenia gravis (OMG) who were treated at the department of strabismus and pediatric ophthalmology in our hospital between July 2018 and July 2022. The control group consisted of patients treated with traditional methods in the neurology department during the same period. The diagnostic criteria for OMG were the presence of feasible ptosis and/or diplopia without limb, bulbar, or respiratory involvement. Auxiliary examinations included thymus computed tomography (CT) scanning, electromyography, and anti-acetylcholine receptor antibody (Anti-AChR-Ab) examination. If the neostigmine test was negative, the diagnosis could only be made if one of the three tests of thymus CT scanning, electromyography, and Anti-AChR-Ab antibody was positive. The exclusion criteria were those who did not support the treatment plan; those who could not complete follow-up on time; and those with other diseases that could not be treated with this treatment scheme. This study adhered to the Declaration of Helsinki, and ethical approval for the study was obtained from the local ethics committee. A detailed medical history was obtained from each patient who also received general and ocular examinations at same time, including demographics, clinical and neuro-ophthalmologic findings, and disease duration. In addition to the neostigmine test, several examinations were also performed, including electrophysiologic tests (such as repetitive nerve stimulation (RNS), and single-fiber electromyography (EMG)), CT scans of the chest, anti-AChR-Ab test, routine blood and urine tests, and lab tests for blood sugar, blood lipid, liver and kidney function, thyroid function, erythrocyte sedimentation rate, rheumatoid factor, and C-reactive protein. Each patient also underwent examinations for slit lamp, fundus and visual acuity, and random intraocular pressure measured based on the time of visit using a non-contact tonometer.

Detailed information on patient’s condition and different treatment methods with their advantages and disadvantages were provided to the enrolled patients and their families prior to formal treatment. All patients or their parents signed a written informed consent form approved by the institutional review board, with their understanding of the risks involved. Explicit written informed consent to publish all data related to the study, including individual details, images, and videos, was also obtained from the patients or their parents (for children < 18 years of age).

### Evaluation criteria

The outcome of MG patients at the last visit was determined based on Maximum Myasthenia Gravis Foundation of America (MGFA) postintervention score(PIS) [22]. Their MGFA-PIS favorable outcomes were also evaluated, including complete stable remission (CSR), pharmacologic remission (PR), and minimal manifestations Status (MMS) for 1 year, while unfavorable outcomes were the following status of MG, such as improved, unchanged, worse, exacerbation, and death.

In the periocular injection group, when evaluating their response to initial treatment, the degree of ptosis and extraocular muscle paralysis in OMG patients was recorded in detail, including the grade of ptosis, according to a scale ranging from 0 to -4, where 0, normal; -1, recognizable with careful observation; -2, evident ptosis, upper eyelid margin covering < 1/2 cornea, no significant impact on vision; -4, completely unable to open the eyelid fissure, -3,between − 2and − 4; As well as the grade of ocular motor duction, based on a scale ranging from 0 to -5, where 0, normal; -5, lack of muscle function; the range from − 1 to -4, not reaching the midline, in 25% increments.

The observed indicators in the control group (traditional oral administration) were the rate of achievement of MMS or better, including (optosis) not affecting vision at primary position, and (ophthalmoplegia) an absence of diplopia at primary position. However, the specific degree of ptosis and ophthalmoplegia was not evaluated in the control group.

### Treatment

This study aimed to evaluate the efficacy of local injections of triamcinolone acetonide compared to oral medication in treating OMG. The patients in ocular injection group received weekly injections of 20 mg triamcinolone acetonide (Zhejiang Xianju Pharmaceutical Co., Ltd, 40 mg) in the orbit of the affected eye, but those in the control group only received oral medication, including pyridostigmine and prednisone. Briefly, after preparation of diluted solution of triamcinolone acetonide (40 mg in 1 ml sterile water), 0.5 ml of this solution was slowly injected inside the orbital rim of the superior lateral quadrant or superior internal quadrant, posterior to the orbital septum. Changes in ptosis and extraocular muscle paralysis were observed and recorded after treatment. In case of multiple muscles were found to be paralyzed, the most severe degree of paralysis was recorded. Once the symptoms of ophthalmoplegia were relieved, the injection frequency was modified as once a month until total period of 6 months. If symptoms did not improve after 4 injections, or if at any time the patient’s condition became worse, they were transferred to a neurologist for treatment. Patients continued to be followed up every month after stopping treatment. If the patient had uncomfortable symptoms, they were advised to see a doctor at any time. If the condition relapsed after stopping treatment, local injection of triamcinolone acetonide was given again.

The treatment goal was to achieve the MGFA-PIS classification MMS or better, with no more than grade 1 medication side effects according to Common Terminology Criteria for Adverse Events (CTCAE) [4]. If the treatment goals could not be achieved, systemic medication pyridostigmine or /and tacrolimus should be added.

Meanwhile, OMG patients in the control group were given traditional oral treatment by the methods of MGFA guidelines and specific treatment methods as follows. For the patients only with ptosis, they were given pyridostigmine (average dosage: 60 mg 2–5 times daily), while for those who remained symptomatic on pyridostigmine or had not met treatment goals, corticosteroid (prednisone) was needed. For the patients with ophthalmoplegia, combination therapy with pyridostigmine and prednisone was given initially. In detail, prednisolone 0.5–1 mg/kg was given with an initial target dosage of 15–20 mg daily and increased as 5 mg every three days, with its peak dose 40-60 mg per day, which will be gradually reduced to 10 mg per week after 4–8 weeks according to changes in the patient’s symptoms. If corticosteroids are refused, contraindicated, or in the case of the ongoing high-dose greater than 10 mg/day, nonsteroidal immunosuppressive therapies, such as tacrolimus, 1.5 mg, bid, were required. Patients in the control group were followed up every week, except for those with stable conditions who were followed up every 4 weeks.

Each patient was followed up for 6 months. Their symptoms of ptosis, eye movements, diplopia, and other systemic symptoms, as well as the treatment-related adverse reactions were recorded.

### Statistical methods

The statistical analysis used T-test (SPSS statistical software version 23.0) for comparing the difference in age, time of onset, and follow-up time between the two treatment groups, Wilcoxon’s Sign Rank Test was used to compare the changes in the degree of upper eyelid ptosis and ophthalmoplegia, and Chi-square test for comparing the therapeutic effects on ptosis and ophthalmoplegia before and after treatment in the two groups.

## Results

### Basic information of the OMG patients

During the study period, a total of 25 eligible patients with OMG were enrolled in the ocular injection group (study group), including 3 patients failing to complete follow-up. Therefore, the final number of eligible patients in the study group was 22. Additionally, 38 eligible patients with OMG were included in the control group (traditional oral administration) during the same period. Of the total 60 patients, 23 (39.3%) were male, and 37 (60.7%) were female, with an age range from 13 to 83 years and an average age of 48.6 ± 20.2 years.

Regarding the comorbidities of the patients, the study group included 1 patient with diabetes, 2 with hypertension, and 1 with hyperthyroidism. In the control group, there were 3 cases of hypertension, 1 case of diabetes, 2 cases of hyperthyroidism, 1 case of thyroid tumor surgery, 1 case of decreased thyroid function, 1 case of hyperuricemia, and 2 cases of coronary heart disease. All patients with thyroid disease in this study did not exhibit TAO related symptoms. Based on the presenting ocular signs, patients were categorized into three types: Type A, which included only symptoms of ptosis; Type B, which included symptoms of ptosis and diplopia but no significant limitation of eye movement; and Type C, which included both ptosis and ophthalmoplegia. The basic information of the patients is detailed in Table 1, including the number of cases for each type, the number of patients with monocular or bilateral ptosis, the number of ophthalmoplegic muscles affected, the examination results of the neostigmine test, Ach-R, electromyography, as well as thymus CT, time from onset to presentation, and follow-up time. There were no significant differences in baseline characteristics between the study and control groups.

**Table 1:** Basic information of OMG patients in both the study group and the control group before treatment

	Study Group (22 cases)	Control Group (38 cases)	Statistical values	*P*
Age (years)	49.0 + 19.2	49.5 + 22.6	0.09	0.92
Sex (Male/Female)	18/20	5/17	3.57	0.06
Ocular symptoms (type A/B/C)	2/16/4	12/20/6	4.01	0.13
Ptosis (binocular / monocular / none)	14/8/0	20/18/0	0.68	0.40
Number of paralyzed extraocular muscles (none/1/2/multiple).	6/3/9/4	18/5/11/4	2.62	0.45
Neostigmine test (positive/invisible)	17/5	29/9	0.007	0.93
Anti-achr-Ab (Positive/negative)	11/11	13/25	1.44	0.22
Electromyography (Positive/negative)	10/12	18/20	0.02	0.88
Presence of thymoma (Positive/negative)	3/19	3/35		0.65
Onset time (days)	34.2 + 33.5	28.7 + 19.9	0.71	0.48
Follow-up time (months)	22.2 + 7.53	22.7 + 7.55	0.24	0.81

Type A: Only symptoms of ptosis; Type B: Symptoms of ptosis and diplopia, but no significant limitation of eye movement; Type C: ptosis and ophthalmoplegia. Anti-AChR-Ab: anti-acetylcholine receptor antibody.

### Treatment efficacy

#### Initial treatment response

During the study period, following the administration of four periocular injections of triamcinolone acetonide, it was observed that three out of the 22 eligible patients with OMG did not respond to the initial treatment and were categorized as non-responsive in this study. Subsequently, these patients received alternative treatment in the form of pyridostigmine and/or tacrolimus (traditional drug treatment), which led to a gradual improvement in their symptoms. In contrast, 19 out of the 22 eligible patients with OMG exhibited a positive response to the treatment. Their average degree of ptosis before the initiation of treatment was measured at -3.00 ± 0.69, and after completing four periocular injections, this resulted in an overall cure rate of 86.4% (19 out of 22 patients), meeting the defined treatment goals (degree of ptosis<-2). Notably, these treatment results remained stable for the subsequent three months.

**Fig. 1:**
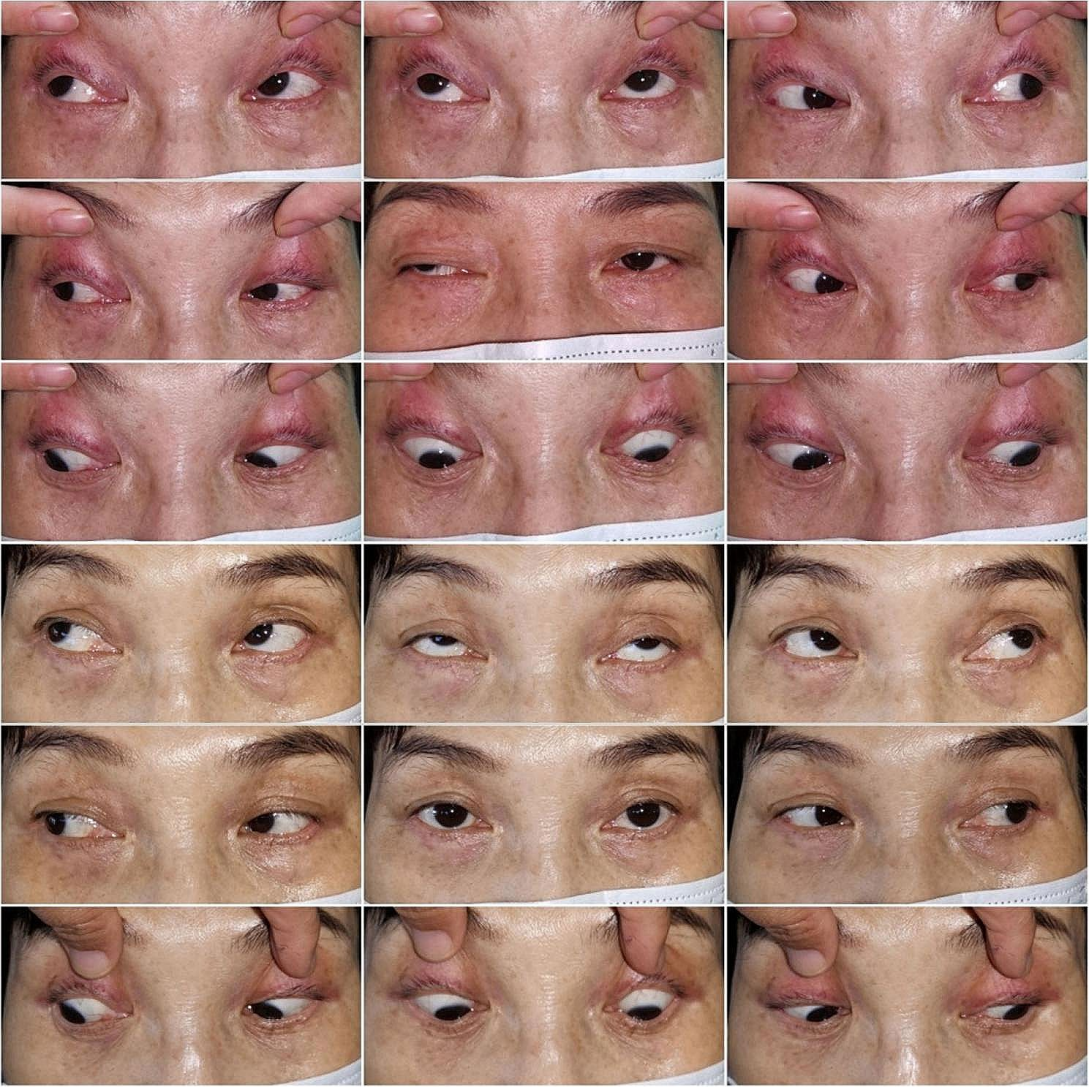
Typical case 1. a 56-year-old female OMG patient with ptosis in the right eye accompanied by exotropia and diplopia, but there is no obvious limitation of eye movement. After two rounds of peribulbar injection of triamcinolone acetonide, her symptoms of strabismus, and diplopia were alleviated with a slight ptosis. From top to bottom, eye position photos before treatment and 2 weeks after treatment

There was a statistically significant change in the degree of ptosis one month after treatment compared to before treatment (Wilcoxon’s Sign Rank Test, Z = 3.89, *P* < 0.001). For 4 patients with intermittent strabismus and diplopia, but without limited eye muscle movement, half cases (2/4) had symptom relief after two injections of triamcinolone acetonide (Fig. 1, Typical case 1), while the remaining two patients had symptom relief after three doses. The number of cases corresponding to the degree of ptosis at different time points is shown in Table 2.

**Table 2:** The number of cases corresponding to the degree of ptosis at different time points after treatment

The degree of ptosis	Before treatment (22cases)	After treatment		
		One-week (22cases)	4-week (22cases)	12-week(19cases)
**0**	0 (0%)	6 (27.3%)	13 (59.1%)	13 (68.4%)
**-1**	1 (4.5%)	5 (22.7%)	4 (18.3%)	4 (21.1%)
**-2**	2 (9.1%)	6 (27.3%)	2 (9.1%)	2 (10.5%)
**-3**	15 (68.2%)	3 (13.6%)	1 (4.5%)	0 (0%)
**-4**	4 (18.2%)	2 (9.1%)	2 (9.1%)	0 (0%)
**Average**	-3.00 + 0.69	-1.54 + 1.29	-0.86 + 1.32	-0.42 + 0.69

**Table 3:** The number of cases corresponding to the degree of Ophthalmoplegia at different time points after treatment

The degree of ophthalmoplegia	Before treatment (16cases)	After treatment		
		One-week (16cases)	4 -week (16cases)	12-week (15cases)
0	0 (0%)	0 (0%)	6 (37.5%)	11 (73.3%)
-1	0 (0%)	2 (12.5%)	7 (43.8%)	3 (20.0%)
-2	3 (18.8%)	8 (50.0)	2 (12.5)	1 (6.7%)
-3	8 (50.0%)	6 (37.5%)	1 (6.3%)	0 (0%)
-4	5 (31.3%)	0 (0%)	0 (0%)	0 (0%)
-5	0 (0%)	0 (0%)	0 (0%)	0 (0%)
Average value	3.12 + 0.72	2.25 + 0.68	0.86 + 0.88	0.33 + 0.62

Among the 16 patients with ophthalmoplegia, 15 had improved symptoms after treatment (Table 3). The patients in the control group were treated with pyridostigmine (for cases only with simple ptosis) or pyridostigmine plus prednisone (ptosis along with ophthalmoplegia or/and diplopia). The achievement rates of treatment goals in those patients with ptosis were 50% and 60.5% at 4 weeks and 12 weeks after treatment, respectively. And in those with ophthalmoplegia, the rates as mentioned above were 30% and 50% at 4 weeks and 12 weeks after treatment, respectively. The difference in treatment response between the study and control groups was statistically significant (*P* < 0.05). Please refer to Table 4 for more detailed information on treatment response.

**Table 4:** The number of cases achieving treatment goals for ptosis and ophthalmoplegia at different treatment time points

Time	Ptosis		*P*	Ophthalmoplegia		*P*
	Study group (22 cases)	Control group (38 cases)		Study group (16 cases)	Control group (20 cases)	
4 weeks	19 (86.3%)	19 (50%)	0.006	12 (75%)	6 (30%)	0.07
12 weeks	19 (86.3%)	23 (60.5%)	0.05	14 (87.5%)	10 (50%)	0.03

Note: In the study group, 3 patients who were treated for 1 month but failed to respond were counted as failing to meet the treatment goals in this table

#### Evaluation at time of last follow-up

**Fig. 2:**
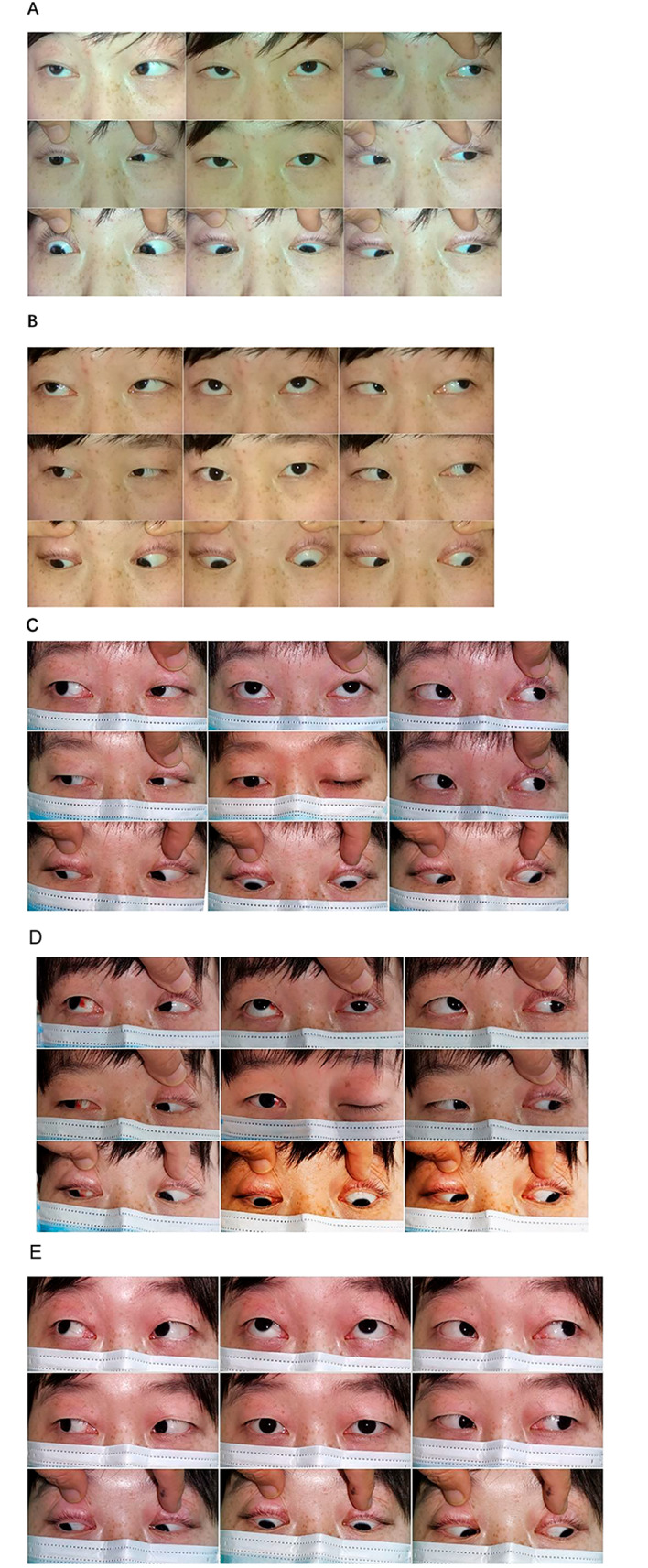
Typical case 2. a 22-year-old female patient with OMG who initially had mild ptosis and abduction in the right eye (Figure **A**). After four injections of triamcinolone acetonide per week, her symptoms were completely relieved (Figure **B**). The treatment was maintained for 3 months and then discontinued medication. Recurrence occurred 3 months after discontinuation, with ptosis of left eye (Figure **C**). The patient’s symptoms rapidly worsened, and developed supraduction and neck weakness. Diagnosis of mild GMG was made (Figure **D**). After re-injection of triamcinolone acetonide with the combination of oral administration of tacrolimus for 6 weeks, her symptoms recovered (Figure **E**) again. At present, MMS condition was maintained using medication. From top to bottom, Figure **A**, **B**, **C**, **D**, **E**

Among the 19 patients in the study group who were relieved by treatment during a period of 22.2 ± 7.53 months, 21.0% (4/19) did not relapse after stopping treatment and met CSR. However, in remaining 15 patients, 9 patients experienced relapse after stopping treatment, 31.6% (6/19) relapsed from 2 to 12 months, and 15.8% (3/19) experienced mild systemic symptoms that transformed into mild GMG. These patients received re-injection treatment, some of which were treated with oral neostigmine (2 cases) and triamcinolone acetonide in combination (2 cases, Fig. 2, Typical case 2), due to poor symptom control. At the time of last follow-up, 42.1% (8/19) in the study group reached PR and 31.5% (6/19) still had symptoms. Additionally, 2 patients with thymoma ultimately received thymectomy due to concerns about drug side effects and other reasons.

Among the 38 patients in the control group, 13.1% (5/38) reached CSR, 39.4% (15/38) experienced relapse, in which 4 patients relapsed more than twice and 9 cases converted to mild GMG. Moreover, 36.8% (14/38) patients in the control group were treated with tacrolimus. One patient with thymoma ultimately underwent thymectomy, because of poor control of symptoms. At the last follow-up, 5 patients still had obvious symptoms of ophthalmoplegia and diplopia, but no other more serious situation had occurred.

Compared with the patients (3/19, 15.8%) who experienced mild systemic symptoms that transformed into mild GMG in the study group, the difference in percentage of the patients with similar situation between the two groups had not statistically significance (Fisher exact test, *P* = 0.13).

Based on the results of the study, it appears that the treatment in the study group was more effective than in the control group in terms of reducing the need for maintenance medication. At the last follow-up, only 10.5% (2/19) of patients in the study group required oral pyridostigmine to maintain symptoms compared to 50% (19/38) in the control group (Fisher exact test, *P* = 0.03) and the specific use of drugs in control group is detailed in Table 5. The final treatment results of the two groups are shown in Table 6.

**Table 5:** Summary of medications used in the control group

Type of medication	Py	Py + Pr	Py + T	Pr + T	Py + Pr + T
Cases (Number, %)	2 (3.3%)	22 (36.7%)	3 (5%)	1 (1.7%)	10 (16.7%)

Py, pyridostigmine; P, Prednisone; T, tacrolimus

**Table 6:** Treatment efficacies at the last follow-up

Treatment result	Study group(22cases)	Control group (38cases)	Statistical values	*P*
CSR	3 (13.6%)	5 (13.2%)		1.00
PR	8 (36.4%)	13 (34.2%)	0.81	1.00
MMS	6 (27.3%)	12 (31.6%)	0.73	0.78
Other cases	5 (22.7%)	8 (21.1%)	0.87	1.00
Relapsed	6 (27.3%)	15 (39.5%)	0.34	0.42
GMG	3(13.6%)	9(23.7%)	0.34	0.51

Other cases: These include patients who do not respond to treatment, do not achieve MMS status, and undergo thymectomy.

### The sources of patients with GMG

Among the patients with GMG, there were 19.4% (7/36) with initial symptoms of ophthalmoplegia and 16.7% (4/24) without initial symptoms of ophthalmoplegia, the difference of which was not statistically significant (chi square 0.78, *P* = 1.0). However, there was a statistically significant difference (Fisher’s exact test, *P* = 0.02) in the development of secondary GMG between patients with anti-AChR-Ab (33.3%, 8/24) and those without anti-AChR-Ab (8.3%, 3/36). Additionally, the study group had a lower rate of secondary GMG (3/19, 15.8%) than the control group (9/38, 23.6%), although this difference was not statistically significant. However, further studies with larger sample sizes are needed to confirm these findings.

### Complications

In terms of complications, in the study group, 5 patients experienced 8 times of intraorbital hematoma caused by injection, which recovered within 1–4 weeks after targeted treatment (local compression and cold compress), and there were no glaucoma or other serious complications. The patient’s intraocular pressure remained stable, without complications, such as secondary glaucoma, cataracts, endophthalmitis, or systemic complications like obesity or impaired glucose tolerance. However, in the control group, 13.1% (5/38) cases experienced headaches, significant weight gain was reported in 60.5% (23/38) of cases, and 10.5% (4/38) of cases had abnormal glucose tolerance, but no other serious complications occurred.

## Discussion

This study is the first to report on the use of peribulbar injection of triamcinolone acetonide for the treatment of ocular myasthenia gravis (OMG) in 22 patients, with a comparison of its effectiveness with conventional oral drug therapy. The study found that, except for three patients who were insensitive to hormones, peribulbar injection of triamcinolone acetonide alone was better and faster at controlling ocular symptoms than traditional oral drugs. However, there was no clear difference in the long-term complete stable remission (CSR) rates or prevention of generalized myasthenia gravis (GMG).

Previous studies have shown that OMG has local pathogenesis involved, despite MG being considered a systemic immune disease in the past. The unique organizational structure of the extraocular muscles (EOM) [15], as well as their unique immune-related gene expression [16], make them more susceptible to autoimmune damage compared to other skeletal muscles. Animal studies have also shown that the neuromuscular connection of EOM is more vulnerable to complement-mediated injury than other muscles, and locally form inflammatory lesions [14, 17]. Therefore, the pathogenesis of OMG has a local mechanism, because of which the lesion is limited to the ocular muscles.

Actually, local administration of corticosteroids by peribulbar injection has been shown to be a viable treatment option for OMG, based on previous studies on TAO [18–20]. The advantage of this approach is that it allows for a higher concentration of the drug to reach the affected ocular muscles, leading to faster and more effective symptom relief. It also has a shorter duration of treatment compared to systemic administration, reducing the risk of side effects associated with long-term corticosteroid use.

Our team has modified the local delivery method for treating OMG by uniformly giving peribulbar injection of triamcinolone acetonide to all patients. This approach seems to have the potential to provide more consistent and effective treatment for OMG patients. It is also worth noting that the previous study using topical corticosteroids showed promising results, suggesting that local administration of steroids could be an effective treatment option for OMG [21]. All the results of our local treatment approach are consistent. However, more research is needed to confirm the effectiveness and safety of this approach for treating OMG specifically.

It seems that there is still limited research on the use of corticosteroids alone for treating OMG, but the existing studies suggest that corticosteroids can be effective in achieving treatment goals and improving symptoms in patients with OMG [10, 23]. The completed randomized placebo-controlled EPITOME (Efficacy of Prednisone for the Treatment of Ocular Myasthenia) trial showed promising results with prednisone treatment, but only 6 patients were enrolled and more research is needed to determine the optimal dosage and duration of treatment, as well as the potential adverse effects of long-term corticosteroid use [10]. Retrospective study also provided some evidence for the effectiveness of corticosteroids in improving specific symptoms such as diplopia and ptosis.

Our results showed that peribulbar injection of triamcinolone acetonide can better and faster alleviate symptoms of ptosis and ophthalmoplegia, compared with the efficacy of traditional oral administration. It is also notable that the different conditions of OMG patients and strict long-term prospective controlled studies make it difficult to judge the efficacy of the therapy with corticosteroids alone [1, 6–8, 24, 25]. Therefore, the patients in the control group were enrolled based on real-world treatment of patients in neurology.

It is important to note that this study has some limitations. The sample size is relatively small, the control group is not ideal as it was not randomized and may not accurately represent the natural course of the disease or the efficacy of other treatments. Additionally, the efficacy of long-term treatment with peribulbar triamcinolone acetonide was not evaluated, and the safety of this treatment modality needs further investigation. Future studies with larger sample sizes, randomized control groups, and longer follow-up periods are needed to confirm the efficacy and safety of peribulbar triamcinolone acetonide as a treatment option for OMG.

It is important to note that not all patients may respond to corticosteroid therapy, whether it is administered systemically or locally [26, 27]. This may be due to individual differences in disease severity or patient characteristics, such as age or comorbidities. It is also possible that non-response is related to underlying immune or inflammatory mechanisms that are not targeted by corticosteroid therapy. In control group in this study, in addition to using corticosteroids, patients also used pyridostigmine and immunosuppressants, making it impossible to observe patients who are not sensitive to corticosteroids. If this aspect is removed, the results of the local treatment group may be better.

Unlike pyridostigmine that is an acetylcholinesterase inhibitor and can provide rapid relief, but not improve prognosis, corticosteroids can improve symptoms, but also improve prognosis [6, 28, 29]. On the other hand, the use of corticosteroids alone may result in adverse events, especially at higher doses (20 mg prednisone daily), such as glucose intolerance or diabetes, hypertension, weight gain, and osteoporosis. A retrospective study showed that the remission rate for long-term symptoms in patients who used low doses of corticosteroids was around 46%, nearly 37% of all patients required addition of immunosuppressants to manage their OMG symptoms and/or reduce adverse events related to corticosteroids [30]. Additional treatment with immunosuppressants can help manage symptoms and reduce adverse events, leading to a higher remission rate [6, 30].

Overall, the results of this study suggest that local injection of corticosteroids can effectively improve the symptoms of OMG, including secondary GMG [7] and can reduce the need for other medications, such as pyridostigmine, in the long term. Additionally, the use of corticosteroids and immunosuppressants may help to reduce the risk of developing GMG, a more severe form of the disease [8, 25, 29].

Our results showed that during the follow-up period, 3 out of 22 patients in the study group developed GMG, with an incidence rate of 13.6%, compared to 9 out of 38 patients in the control group, with an incidence rate of 23.7%. It’s possible that the lack of statistical significance in the difference in secondary GMG incidence between the study group and control group may be due to the small sample size. It’s important to note that larger studies are needed to confirm whether the lower incidence rate of secondary GMG in the study group is significant. Nonetheless, the better early symptom control seen in the study group may have contributed to reducing the risk of secondary GMG, as rapid and effective control of symptoms is believed to be a key factor in preventing disease progression [9].

There are several risk factors that may affect the generalization of OMG, including patient’s age of onset, degree of initial symptoms, anti-AChR-Ab positive rate, and thymoma [9, 12, 13, 26, 31]. Among those risk factors, we found that the concentration of anti-AChR-Ab is related to the generalize conversion rate. Our finding is consistent with previous research.

Although complications with periocular injections of steroids are not common, they can still occur, such as globe perforation, arterial occlusion, toxic optic neuropathy, or atrophy of subcutaneous tissue in the face [19], all of which should be taken seriously. Patients should be closely monitored for any signs of complications, and healthcare providers should follow proper injection techniques to minimize the risk of complications. In this study, the patient’s intraocular pressure was stable during the treatment period. There were no other complications, except for intra frame hematoma, indicating that local medication is safer than systemic medication. However, local pain and patient fear caused by injection are important factors that affect patients’ long-term adherence to treatment. To this end, it is necessary to provide them with education and support to address any concerns or fears they may have about injections. Sustained-release agents with longer therapeutic durations may also be a viable option for improving patient acceptance of treatment. Ultimately, the choice of treatment should be based on individual patient factors and preferences, as well as careful consideration of the potential benefits and risks of each treatment option.

Conclusion: based on the study’s findings, it seems that repeated peribulbar injection of triamcinolone acetonide can be an effective treatment for initial symptoms of ocular myasthenia gravis (OMG) and may have the potential to reduce the use of other drugs, thereby minimizing associated side effects. Additionally, it may reduce the risk of secondary generalized myasthenia gravis (GMG) in the long run. However, further research is needed to confirm the long-term efficacy of this treatment and to explore any potential complications associated with long-term local hormone injection.

## Data Availability

The authors confirm that the data supporting the findings of this study are available within the article. More detailed raw data are available from the corresponding author upon reasonable request.

## Abbreviations

OMG: Ocular myasthenia gravis
MG: Myasthenia Gravis
GMG: Generalized myasthenia gravis
EOM: Extraocular muscle
TAO: Thyroid Associated Ophthalmopathy
Anti-AChR-Ab: Anti-acetylcholine receptor antibody
RNS: Repetitive nerve stimulation
EMG: Single-fiber electromyography
CT: Computed tomography
MGFA: Maximum Myasthenia Gravis Foundation of America
PIS: Postintervention score
CSR: Complete stable remission
PR: Pharmacologic remission
MMS: Minimal manifestations Status
CTCAE: Common Terminology Criteria for Adverse Events
